# Maternal markers for detecting early-onset neonatal infection and chorioamnionitis in cases of premature rupture of membranes at or after 34 weeks of gestation: a two-center prospective study

**DOI:** 10.1186/1471-2393-11-26

**Published:** 2011-04-07

**Authors:** Thomas Popowski, François Goffinet, Françoise Maillard, Thomas Schmitz, Sandrine Leroy, Gilles Kayem

**Affiliations:** 1 Epidemiological Research Unit on Perinatal and Women's Health, INSERM U953, Paris, France; 2 Department of Obstetrics and Gynecology, Maternity Port-Royal, Cochin-Saint Vincent-de-Paul Hospital, Paris, France and University Paris V, Paris, France; 3 Department of Obstetrics and Gynecology, Hôpital Robert Debré, Paris, France and University Paris VII, Paris, France; 4 Centre for Statistics in Medicine University of Oxford, Oxford, UK; 5 Department of Obstetrics and Gynecology, CHI Creteil, Creteil, France

## Abstract

**Background:**

Accurate prediction of infection, including maternal chorioamnionitis and early-onset neonatal infection, remains a critical challenge in cases of preterm rupture of membranes and may influence obstetrical management. The aim of our study was to investigate the predictive value for early-onset neonatal infection and maternal histological and clinical chorioamnionitis of maternal biological markers in routine use at or after 34 weeks of gestation in women with premature rupture of membranes.

**Methods:**

We conducted a two-center prospective study of all women admitted for premature rupture of membranes at or after 34 weeks of gestation. The association of C-reactive protein, white blood cell count, vaginal sample bacteriological results, and a prediction model at admission, for early-onset neonatal infection and maternal chorioamnionitis were analyzed by comparing areas under the receiver operating characteristic curves and specificity.

**Results:**

The study included 399 women. In all, 4.3% of the newborns had an early-onset neonatal infection and 5.3% of the women had clinical chorioamnionitis. Histological chorioamnionitis was detected on 10.8% of 297 placentas tested. White blood cell counts and C-reactive protein concentrations were significantly associated with early-onset neonatal infection and included in a prediction model. The area under the receiver operating characteristic curve of this model was 0.82 (95% CI [0.72, 0.92]) and of C-reactive protein, 0.80 (95% CI [0.68, 0.92]) (p = 1.0). Specificity was significantly higher for C-reactive protein than for the prediction model (48% and 43% respectively, p < 0.05). C-reactive protein was associated with clinical and histological chorioamnionitis, with areas under the receiver operating characteristic curve of 0.61 (95% CI [0.48, 0.74]) and 0.62 (95% CI [0.47, 0.74]), respectively.

**Conclusions:**

The concentration of C-reactive protein at admission for premature rupture of membranes is the most accurate infectious marker for prediction of early-onset neonatal infection in routine use with a sensitivity > 90%. A useful next step would be a randomized prospective study of management strategy comparing CRP at admission with active management to assess whether this more individualized care is a safe alternative strategy in women with premature rupture of membranes at or after 34 weeks.

## Background

Premature rupture of membranes (PROM) occurs in 8% of pregnancies, 3% before and 5% after 37 weeks of gestation [1]. Accurate prediction of infection, including maternal chorioamnionitis and early-onset neonatal infection (EONI), remains a critical challenge in these cases. EONI, generally acquired prenatally in pregnancies with PROM, is the most serious consequence of maternal infection and is associated with increased neonatal morbidity and mortality [2,3].

Numerous studies in recent years have failed to identify a satisfactory prenatal marker of infection to predict maternal chorioamnionitis and EONI. For these reasons, guidelines for the management of women with PROM do not take prenatal markers of infection into account but are usually based on the gestational age at which PROM occurs [4,5].

Many obstetricians therefore consider active management, usually defined as the induction of labor in the 12 hours after PROM, as the best strategy for preventing chorioamnionitis and EONI in cases of PROM at or after 34 weeks [4,5]. Although a few studies have found systematic active management at or after 34 weeks to be advantageous for infectious outcomes [6,7], the choice between active or expectant management at 34 to 36 weeks continues to be controversial because this strategy may increase early delivery, which has been associated with increased neonatal morbidity, behavioral disorders, and health-care costs [8-12]. Furthermore, regardless of gestational age, induction of labor is sometimes contraindicated for various reasons or situations, including previous cesarean delivery and breech presentation. Active management in these cases would increase the rate of cesarean delivery.

The use of maternal laboratory markers at or after 34 weeks of gestation would help to distinguish women at risk from those who do not require active management, that is, for whom pregnancy can safely be prolonged. Their use might make it possible to await spontaneous labor and vaginal delivery and thus avoid cesareans when induction is contraindicated or unsuccessful but vaginal delivery remains appropriate. Prenatal maternal markers of infection at or after 34 weeks have, however, been insufficiently studied. Those that might be easily used in routine care are serum C-reactive protein (CRP) levels, white blood cell counts (WBC count), and bacterial analysis of vaginal samples. No study has included enough women at or after 34 weeks of gestation to allow the predictive values of these markers to be estimated.

Thus, our main objective was to investigate the predictive value for EONI and maternal histological and clinical chorioamnionitis of CRP, WBC count, and the bacteriological analysis of vaginal samples in routine use at or after 34 weeks of gestation in women with PROM and thus provide a safe alternative to systematic active management.

## Methods

### Study design

This prospective hospital-based study conducted from January 2004 through February 2006 in two French tertiary university referral centers investigated the predictive value of maternal serum and vaginal markers for clinical and histological chorioamnionitis and for EONI. This analysis included all women with PROM at or after 34 weeks of gestation in singleton pregnancies, except those in spontaneous labor at admission and those who gave birth more than 72 hours after admission. These women were excluded to preserve the physiological temporal effect between infectious markers at admission and maternal-fetal infection. Clinical PROM was confirmed by an immunochromatographic dipstick test that uses monoclonal antibodies to detect IGFBP-1 from amniotic fluid.

A standardized form was used to collect data prospectively at admission and thereafter. The attending physician obtained informed consent from all participants. The study was approved by the relevant institutional review board (CPP Ile de France).

Maternal serum samples were taken at admission from all women. Antibiotic treatment was started at admission for women if the third-trimester vaginal sample contained *group B Streptococci*, and they were not expectantly managed. Antibiotic treatment for all other women began 12 hours after PROM. Amoxicillin was administered except in cases of penicillin allergy, when erythromycin was used instead. Women with a clinical infection at admission were not included in the study. One center used active management, defined by induction of labor or cesarean delivery when induction was contraindicated, in the 12 hours after PROM. The other center used expectant management until 37 weeks, including clinical and laboratory monitoring for infection; it also used expectant management at or after 37 weeks for 48 hours after PROM, to promote spontaneous vaginal delivery when possible and not contraindicated.

This study was submitted to the French Ethical Review Committee and was found to be in conformity with the laws and regulations of the country in which the research experiment was performed.

### Outcome definitions

Three infectious outcomes were studied: EONI, clinical chorioamnionitis and histological chorioamnionitis.

EONI was defined by the pediatrician as a neonatal infection within 72 hours after birth [13]. Confirmed neonatal infections were defined by a positive blood culture or a positive cerebrospinal fluid culture associated with clinical signs of infection. Probable neonatal infection was defined by clinical signs and a neonatal CRP ≥ 10 mg/L. The final diagnosis of EONI was determined after re-evaluation of the course of laboratory and clinical markers through discharge.

Clinical chorioamnionitis was diagnosed by the attending consultant physician responsible for management from admission through delivery, according to the following criteria: temperature greater than 37.8°C in a gravid patient without evidence of urinary, respiratory, or other localized infection, and any two of these other criteria: either uterine tenderness or foul-smelling amniotic fluid, maternal tachycardia greater than 120 beats per minute, and fetal tachycardia, greater than 160 beats per minute [14].

Acute histological chorioamnionitis was defined as mild or severe acute inflammatory changes in any relevant tissue sample (amnion, chorion, decidua, umbilical cord, or chorionic plate). For the histological analysis of the placenta, tissue samples from the umbilical cord, chorionic plate, and placental membranes were fixed in 10% neutral buffered formalin and embedded in paraffin. Sections of tissue blocks were stained with hematoxylin and eosin. The pathologists in each center, blinded to the clinical information, performed these examinations and classified acute inflammation as minor, mild, or severe, on the basis of the criteria of Salafia *et al *[15]. The histological analysis of the placentas was standardized before the beginning of the study by developing a common analysis protocol.

### Predictive factors

Serum samples taken at admission were used to measure the maternal WBC count (expressed as cells × 10^9^/L) and CRP concentration (expressed as mg/L, assessed by immunoturbidimetry). A vaginal swab sample was also cultured to detect the standard pathogenic genital bacteria according to groups II and III of a French classification (Additional file 1) [16,17].

Finally, we collected data about potential confounding factors, including gestational age (in weeks of gestation at admission), antibiotic prescription at admission, and type of management (expectant or active). Active management was defined as systematic delivery at admission, regardless of gestational age, infectious status, or medical history. Expectant management was defined as any other management, including close monitoring for infectious status, especially for women at 34 to 37 weeks of gestation.

### Statistical Analysis

We first described the population's characteristics and then analyzed the relations between each of the three infectious outcomes (EONI and clinical and histological chorioamnionitis) and all the predictive factors with a logistic regression model. When the relation between the outcome and continuous variables (CRP and WBC count) was not linear, the variables were transformed into fractional polynomials of the lowest possible degree [18]. Binary variables were coded as follows: abnormal bacterial colonization of the genital tract: yes (1), no (0); antibiotic prescription at admission (1), no antibiotic (0); expectant management (1), and active management (0). Because the model was not convergent when gestational age was a continuous variable, it was dichotomized and coded as it usually is in the literature: (1) for gestational age at admission < 37 weeks gestation, (0) for ≥ 37 weeks. We first selected predictors that were significantly associated with infectious outcome in the univariate analysis, with a p value < 0.05. We then used a backward stepwise technique, setting a p value > 0.1 for removal from the model and calculating maximum likelihood ratio estimates. The model's ability to discriminate between women with clinical chorioamnionitis, those with histological chorioamnionitis, and those with infants with EONI, compared with those without identified infection, was evaluated by the area under the ROC curve and compared with that of the continuous variables that remained significant in the multivariable analysis. We next transformed the continuous probability of the outcome given by the model into a binary test, choosing a threshold based on the area under the ROC curve that provided the best specificity with at least 90% sensitivity. The performance of the model was determined by calculating sensitivity, specificity, and positive and negative likelihood ratios.

Internal cross-validation of the final model was performed by bootstrapping (100 times), a computer-intensive statistical method and resampling technique based on random samples of observations to estimate error by systematically recomputing the statistics while omitting one observation at a time from the sample set [19]. Using the same pre-specified 90% sensitivity, we dichotomized continuous predictors into binary variables and used a paired χ^2 ^test to compare their discriminative abilities to those of the model.

Statistical analyses were performed with Stata 10/SE software (StataCorp, College Station, TX, USA) and Confidence Interval Analysis software (London, UK) [20].

## Results

**Figure 1:**
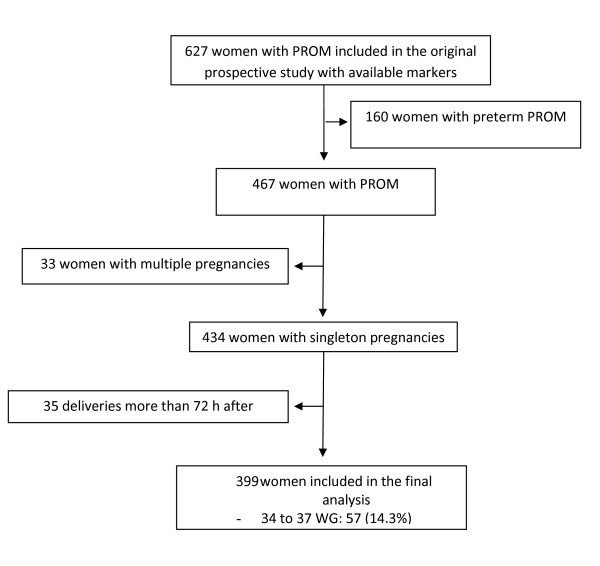
**Flow chart of the study population**. Abbreviations: PROM, Premature rupture of membranes; WG, Weeks of gestation.

During the study period, 627 women were admitted for PROM, 434 of them at or after 34 weeks of gestation (Figure 1). Among the latter, 399 gave birth within 72 hours after admission and were therefore included in the final analysis; 57 (14.3%) had PROM before 37 weeks, 21 (5.3%) had clinical chorioamnionitis, and 32 histological chorioamnionitis (10.8% of the 297 women for whom histological analysis was available). EONI was diagnosed for 17 (4.3%) newborns, including six (35.3%) whose mothers had clinical chorioamnionitis and six (35.3%) whose mothers had histological chorioamnionitis (including one whose mother had both). Mean gestational age at PROM was 38.5 weeks. The characteristics of women and their babies are summarized in Table 1. Overall, 128 women (32.1%) had active management and 271 (67.9%) expectant management, that is, spontaneous deliveries. Among the latter, 211 (78%) took place within 12 hours after admission. Overall, only 57 of the 399 women (14%) were delivered at 34-36 weeks: one had clinical chorioamnionitis, five histological chorioamnionitis, and 2 had a neonate with an EONI. These figures are unfortunately too low to allow a useful statistical analysis for this subgroup.

**Table 1:** Maternal and neonatal characteristics

Women	N = 399
Maternal age, years (mean +/- [SD])	31.7 +/- [5.5]
Nulliparous, n (%)	214 (53.8)
Gestational age at inclusion in study (weeks) (mean +/- [SD])	38.5 +/-[1.6]
Time elapsed from admission to delivery (hours) (mean +/- [SD])	25 +/- [15]
Active management, n (%)	128 (32.1)
Expectant management, n (%)	271 (67.9)
Histological chorioamnionitis, n (%) (N = 297)	32 (10.8)
Funiculitis, n (%) (N = 297)	14 (4.7)

**Neonates**	N = 399

Birth weight (grams) (mean +/- [SD])	3240 +/- [471]
Early-onset neonatal infection, n (%)	17 (4.3)
5-minutes Apgar score < 7, n (%)	14 (3.5)
Transfer to neonatal intensive care unit, n (%)	14 (3.5)

As expected, spontaneous delivery rates differed according to center. In the center that provided an expectant management policy for 48 hours for women after 37 weeks, 76% of the women gave birth after spontaneous delivery, but only 56% of the women in the center with active management, all of those within the first 12 hours after PROM. Neither EONI (p = 0.12) nor clinical chorioamnionitis (p = 0.30) rates differed between the two centers. Histological chorioamnionitis was observed more often in the center with expectant compared with active management (p = 0.042). The cesarean rates were 18.0% (42/233) and 19.3% (32/166) (p = 0.75).

Table 2 reports the relations between infectious outcomes and all predictive factors. We observed no confounding factors; in particular, neither gestational age nor type of management was significantly associated with any of the three infectious outcomes. Because the association between WBC count and EONI was not linear (p = 0.004), we studied it by transforming the WBC count into a first-degree fractional polynomial function. In a univariate analysis, WBC count, CRP, and abnormal bacterial colonization of the genital tract were all associated with EONI. In a multivariable analysis after stepwise reduction, WBC count and CRP remained significantly associated with EONI (Table 2). The model fit was good (p = 0.54, Hosmer and Lemeshow test).

**Table 2:** Crude and adjusted association between EONI, clinical and histological chorioamnionitis and gestational age at delivery, antibiotics at admission, management of delivery, and routinely used infectious markers.

	Early Onset Neonatal Infection		Clinical Chorioamnionitis		Histological Chorioamnionitis	
**Binary Variables**	**OR** **[95% CI]**	**Adjusted OR** **[95% CI]^a^**	**OR** **[95% CI]**	**Adjusted OR** **[95% CI]^a^**	**OR** **[95% CI]**	**Adjusted OR** **[95% CI]^a^**

*Gestational Age*						
< 37	0.8 [0.2, 3.6]	-	0.3 [0.0, 2.2]	-	1.1 [0.4, 3.1]	-
≥ 37	1		1		1	

*Antibiotic at admission*						
Yes	0.7 [0.3, 2.1]	-	0.5 [0.2, 1.5]	-	0.6 [0.2, 1.3]	-
No	1		1		1	

*Management of delivery*						
Expectant	0.4 [0.1, 1.6]	-	0.6 [0.2, 1.8]	-	1.6 [0.8, 3.5]	-
Active	1		1		1	

*Abnormal bacterial colonisation, genital tract*						
Yes	2.7 [1.0, 7.2]	2.2 [0.7, 6.6]	1.8 [0.7, 4.6]	-	1.2 [0.5, 2.7]	-
No	1	1	1		1	

**Continuous variables**^b^	**Coefficient** **[95% CI]**	**Adjusted Coefficient** **[95% CI]^a^**	**Coefficient** **[95% CI]**	**Adjusted Coefficient** **[95% CI]^a^**	**Coefficient** **[95% CI]**	**Adjusted Coefficient** **[95% CI]^a^**

*CRP(mg/l)*	0.064 [0.035, 0.093]	0.055 [0.024, 0.086]	0.039 [0.016, 0.062]	0.039 [0.016, 0.062]	0.051 [0.022, 0.080]	0.051 [0.022, 0.080]

*WBC (giga/l)*	0.015 [0.007, 0.023]	0.013 [0.003, 0.022]^c^	0.0001 [-0.0001, 0.0002]	-	0.0001 [0.00003, 0.0002]	-

^a ^Adjusted OR and coefficients for non-significant variables taken from the model just before their elimination after stepwise reduction; those for

significant variables come from the final model

^b ^Coefficients for CRP and WBC were calculated from the logistic regression equation.

^c ^The FP1 transformation used for WBC because of its non-linear association with EONI was: [(WBC/10000)^7.6354^-1.660493995].

**Figure 2:**
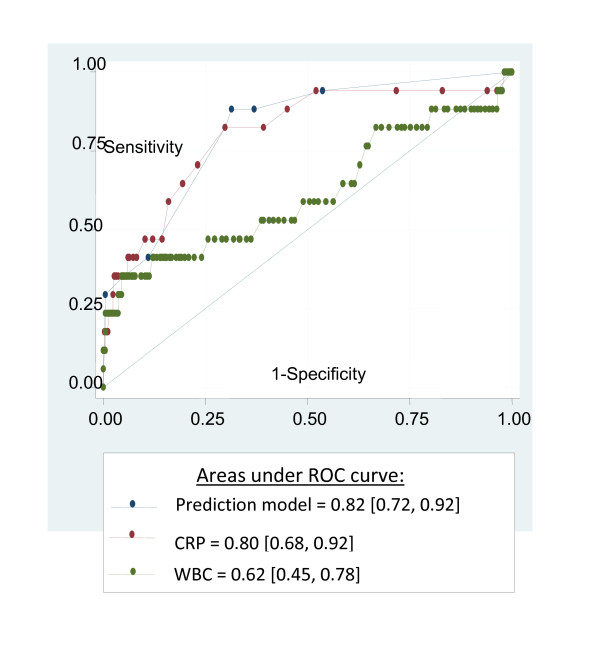
**Comparison of ROC curves of prediction model, maternal serum CRP, and WBC for predicting EONI**.

Using the coefficients assigned to each predictor in the logistic regression model, we derived a prediction model as follows: logit (predicted probability of infant with EONI) = -4.047 + 0.055*CRP + 0.013*[(WBC count/10000)^7.6354 - 1.660, where EONI was coded (0) if absent and (1) if present. The area under the ROC curve of the EONI prediction model was significantly higher than that of WBC count (0.82, 95% CI [0.72, 0.92] vs 0.62 [0.45, 0.78], p = 0.003) but not than that of CRP (0.82 [0.72, 0.92] vs 0.80 [0.68, 0.92], p = 1.0) (Figure 2).

Next, we used ROC curves to define the threshold corresponding to our pre-specified sensitivity of at least 90%, which allowed us to transform the continuous probability of EONI into a binary rule. A positive result according to this rule (corresponding to a predicted probability ≥ 0.022) was significantly associated with EONI (OR = 12.0 [1.6, 91.7]) and had a sensitivity of 94% (77, 99%), a specificity of 43% (38, 47%), a positive likelihood ratio of 1.6 (1.5, 1.9), and a negative likelihood ratio of 0.1 (0.1, 0.7) for the prediction of EONI (Table 3).

**Table 3:** Predictive value of the model and CRP for EONI.

	Prediction Model (≥ 0.022265) [95% CI]	CRP (≥ 5 mg/l) [95% CI]
**Sensitivity**	**94 **[77-99]	**94 **[73-99]
**Specificity**	**43 **[38-47]	**48 **[43-53]
**PPV**	**7 **[5-10]	**7 **[5-12]
**NPV**	**99 **[97-100]	**100 **[97-100]
**Positive LR**	**1.6 **[1.5-1.9]	**1.8 **[1.6-2.1]
**Negative LR**	**0.1 **[0.1-0.7]	**0.1 **[0.0-0.8]
**OR**	**12.0 **[1.6-91.7]	**14.7 **[1.9-112.2]

LR, likelihood ratio

NPV, negative predictive value

PPV, positive predictive value

OR, odds ratio

**Figure 3:**
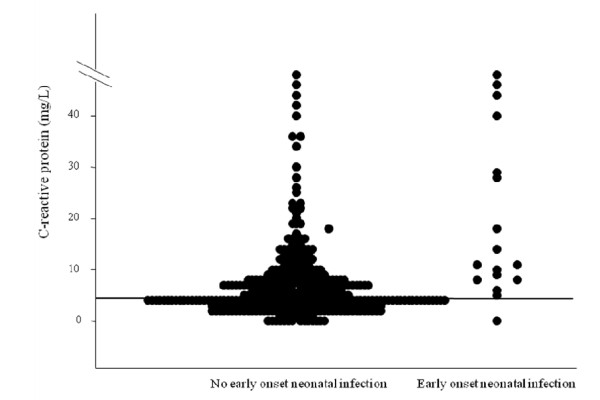
**Distribution of EONI as a function of continuous CRP (horizontal line: CRP = 5 mg/l)**.

We applied the same pre-specified constraint of a sensitivity of at least 90% to dichotomize the only significant independent predictor, CRP, at a threshold of 5 mg/L. Elevated CRP (≥ 5 mg/L) was then significantly associated with EONI (OR = 14.7 [1.9, 112.2]), with a sensitivity of 94% (73-99), a specificity of 48% (43-53), a positive likelihood ratio of 1.8 (1.6, 2.1), and a negative likelihood ratio of 0.1 (0.0, 0.8) for the prediction of EONI (Table 3 and Figure 3). Lastly, the specificity of CRP alone was significantly higher than that of the prediction model (significant difference of 5% (2, 8%)). Internal cross-validation by bootstrapping showed that dichotomized CRP had a stable predictive value for EONI, with a sensitivity of 94% (82, 100%) and a specificity of 47% (42, 54%). The results for the prediction model were also stable after internal cross-validation, with a sensitivity of 94% (82, 100%) and a specificity of 43% (39, 47%).

**Figure 4:**
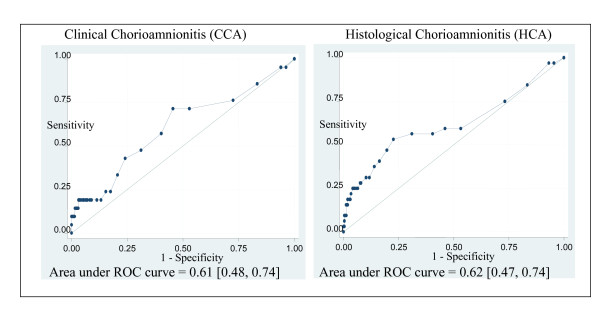
**ROC curves of maternal serum CRP for predicting clinical and histological chorioamnionitis**.

CRP was the only factor significantly associated with both clinical and histological chorioamnionitis, and it was linearly associated with these outcomes. Areas under the ROC curves were 0.61 (0.48, 0.74) for clinical and 0.62 (0.47, 0.74) for histological chorioamnionitis (Figure 4). With a cut-off of 5 mg/L, the predictive values of CRP for clinical chorioamnionitis were 71% (48, 89%) (sensitivity) and 47% (42, 52%) (specificity), with positive and negative likelihood ratios of 1.4 (1.0, 1.8) and 0.6 (0.3, 1.2). For histological chorioamnionitis, sensitivity was 59% (41, 76%), and specificity 47% (41, 53%), with positive and negative likelihood ratios of 1.1 (0.8, 1.5) and 0.9 (0.6, 1.4).

## Discussion

Of the routinely tested prenatal markers, a CRP concentration of 5 mg/L or more was the most accurate predictor of EONI, with a sensitivity of 94% and a specificity of 47%. Its predictive value was not improved by including it in a prediction model with WBC count. A CRP concentration of 5 mg/L or more was also associated with both clinical and histological chorioamnionitis, but its predictive values for both outcomes were low.

This study is, to our knowledge, the first to investigate prenatal markers in women with PROM at or after 34 weeks of gestation and the largest prospective cohort study of serum infectious markers in PROM. In a retrospective study including 90 women with PROM from 23 to 41 weeks of gestation, Yoon *et al*. did not find that either CRP or WBC count had a high predictive value for histological chorioamnionitis [21].

The ability to predict EONI is a high priority for physicians managing women with PROM, because it is the main cause of neonatal morbidity and mortality when PROM occurs at or after 34 weeks [2,3]. We used a pre-specified high sensitivity because the aim of this study was to select a population with a very low risk of infection, who could safely avoid systematic active management. The negative predictive value of the test is important, regardless of its specificity. In fact, systematic active management is a strategy with 0% specificity; compared to it, a specificity of 47% is a good result, with nearly half the women safely able to avoid systematic active management.

The choice between immediate active or expectant management for women with PROM from 34 to 37 weeks of gestation remains controversial [6,7,22]. Guidelines in many countries advocate active management, mainly because of the risk of neonatal infection [4,5]. This strategy, however, may increase the rate of moderately preterm birth, which is associated with significant neonatal morbidity [10-12]. It may also increase the cesarean rate among women for whom induction of labor is contraindicated (or unsuccessful). A survey, published in 2004 and including 508 specialists from all 50 of the United States and 13 other countries, showed that the gestational age at which expectant management is rejected in women with preterm PROM varied significantly between respondents: ≥ 34 weeks for 56%, ≥ 35 weeks for 26%, ≥ 36 weeks for 12%, and ≥ 37 weeks for 4.0% [8]. Canadian and Australian surveys show a similar lack of medical consensus on management from 34 to 36 weeks [9,23].

A recent systematic review included seven randomized controlled trials (690 women) and compared expectant management with early delivery for women with preterm PROM before 37 weeks of gestation (from 25 to 36 weeks) [24]. It identified no difference in neonatal sepsis (RR = 1.3 (0.7, 2.5)) or neonatal respiratory distress (RR = 1.0 (0.7, 1.3)) but found that active management significantly reduced suspected neonatal infection (RR = 0.5 (0.3, 0.8)). Moreover, active management seemed to have no effect on clinical chorioamnionitis (RR = 0.4 (0.2, 1.1)) but was associated with a significant increase in the cesarean rate (RR = 1.5 (1.1, 2.1)) [24]. The authors concluded that there is insufficient evidence about the benefits and disadvantages of immediate delivery, compared with expectant management, for women with preterm PROM. Dare *et al*. conducted a systematic review that finally included 12 randomized or quasi-randomized trials comparing planned early birth with expectant management in women with PROM at or after 37 weeks of gestation. They concluded that clinical chorioamnionitis was significantly less frequent among women with active management compared with those expectantly managed (RR = 0.7 (0.6, 1.0)), but they observed no difference for neonatal infection (RR = 0.8 (0.6, 1.1)) [22]. From our perspective, these findings underline the uncertainty related to PROM management after 34 weeks of gestation and emphasize the need for a predictive strategy to support individualized care based on the individual risk of infection.

Our methodology was selected to reduce bias as much as possible. We analyzed all continuous variables without dichotomizing them to reduce analytic bias [25]. A bootstrap resampling procedure was used to reduce the over-fit of the statistical model on the study population [19]. To prevent any bias related to gestational age, antibiotic prescriptions, or medical management, we adjusted for these factors in our multivariable model. A meaningful temporal relation between infectious markers and the primary outcome was preserved by including only those women who gave birth within 72 hours after admission [26]. The 35 women who delivered more than 72 hours after admission (8.0%) were excluded from the study. This subgroup's characteristics did not differ significantly from those of women finally included in the study and no differences between the groups were observed for EONI (p = 0.69) or for clinical (p = 0.14) or histological chorioamnionitis (p = 0.06). Nonetheless, we did observe a trend to chorioamnionitis in this group with later deliveries, but lack of power prevented a definitive result. Our study shows that a CRP concentration of 5 mg/L or higher predicts EONI with high sensitivity. Measured routinely at admission, CRP may be useful for selecting a population among whom the risk of EONI contraindicates expectant management. In our study, consideration of CRP at admission would have led to active management of 215 women (54%); including 16 whose infants had EONI (94%). The remaining 184 women could have been managed expectantly: most of them (almost 90%) went into labor spontaneously within 72 hours, and only one neonate had EONI [27,28]. Although the systematic active management in one center might be considered a limitation of this study, our results suggest that maternal serum CRP could be a safe alternative strategy to systematic active management, especially in cases of previous cesarean deliveries, or breech presentation, or in women with PROM near term. Given that the strategy of systematic active management has 0% specificity and in view of our findings, nearly half the cases could safely avoid systematic active management. Our results call for a randomized trial, to compare immediate systematic active management in PROM at and after 34 weeks of gestation with management according to maternal serum CRP.

## Conclusions

Maternal serum CRP at admission is the most accurate infectious marker for predicting EONI that is currently in routine use. A useful next step would be a randomized prospective study of management strategy comparing CRP at admission with active management to assess whether this more individualized care is a safe alternative strategy in women with premature rupture of membranes at or after 34 weeks.

## Abbreviations

CI: Confidence interval; CRP: C-reactive protein; EONI: Early-onset neonatal infection; IGFBP-1: Immunoglobulin growth factor binding protein 1; LR: Likelihood ratio; OR: Odds ratio; ROC: Receiver operating characteristics; RR: Risk ratio; WBC: White blood cell;

## Competing interests

The authors declare that they have no competing interests.

## Authors' contributions

TP conducted the analysis and wrote the first draft of the paper. FG contributed to the design of the study and supervised the analysis, writing and editing of the paper. FM assisted with data coding, conducted validation of the data and analysis, and contributed to the writing of the paper. TS assisted with the design of the study and contributed to the analysis and writing of the paper. SL assisted the analysis and contributed to the writing of the paper. GK designed the study, coordinated data collection, determined the data coding, and supervised the analysis, writing and editing of the paper. All authors read and approved the final manuscript

## Pre-publication history

The pre-publication history for this paper can be accessed here:

http://www.biomedcentral.com/1471-2393/11/26/prepub

## Supplementary Material

Additional file 1 **Appendix 1: Vaginal Bacteria with neonatal infection risk**. French classification of pathogenic genital bacteria in function of neonatal infection risk. Click here for file
