# Supplementary material for: Maternal markers for detecting early-onset neonatal infection and chorioamnionitis in cases of premature rupture of membranes at or after 34 weeks of gestation: a two-center prospective study
Source: BMC Pregnancy Childbirth. 2011 Apr 7;11:26. doi: 10.1186/1471-2393-11-26 (PMC3088535; doi:10.1186/1471-2393-11-26)
Supplement: Additional file 1 — Appendix 1: Vaginal Bacteria with neonatal infection risk. French classification of pathogenic genital bacteria in function of neonatal infection risk. [file 1471-2393-11-26-S1.DOC]

**Appendix 1: Vaginal Bacteria with neonatal infection risk.**

Group I:

Usual vaginal bacterial flora without major neonatal risk.

- Lactobacillus (Döderlein flora); usually observed at direct Gram examination (positive Gram bacillus).

Group II :

Vaginal bacterial flora coming from digestive flora with neonatal infection risk.

- Streptococcus agalactiae

- Enterococcus

- Enterobacteria (Escherichia coli, Proteus, Morganella, Klebsiella, Enterobacter and seldom Serratia, Pseudomonas and Acinetobacter)

- Staphylococcus epidermidis and aureus

- Anaerobic bacteria (Bacteroides spp., Prevotella spp., Porphyromonas spp., Fusobacterium spp., Clostridium spp., Peptostreptococcus spp., Veillonella spp., Mobiluncus)

- Gardnerella vaginalis and corynebacteria

- Mycoplasma (particularly Mycoplasma hominis), Ureaplasma urealyticum; and some Haemophilus specifically adapted at genital flora

- Candida albicans

Group III:

Usual bacteria coming from oropharyngeal flora exceptionally and with high risk of neonatal infection in vaginal cavity.

- Haemophilus influenzae and parainfluenzae

- Streptococcus pyogenes

- Streptococcus Pneumoniae

- [Neisseria meningitidis](http://fr.wikipedia.org/wiki/Neisseria_meningitidis)
